# Toxicity Evaluation of Potassium Sorbate In Vivo with Drosophila Melanogaster

**DOI:** 10.3390/insects15090703

**Published:** 2024-09-14

**Authors:** Xubo Zhang, Qian Zhang, Xiaoxuan Song, Wanchen Yang, Andi Cheng, Jianzhen Zhang, Wei Dong

**Affiliations:** Shanxi Key Laboratory of Nucleic Acid Biopesticides, Research Institute of Applied Biology, Shanxi University, Taiyuan 030006, China

**Keywords:** potassium sorbate, *Drosophila*, midgut, cell apoptosis, mitophagy, differentiation

## Abstract

**Simple Summary:**

Potassium sorbate (PS) is widely utilized as a food preservative. In this study, *Drosophila melanogaster* was employed as a model organism to assess the potential toxicity of PS. We examined the impacts of PS on several physiological parameters and discovered that higher levels of PS intake significantly affected the majority of these parameters. Additionally, our data suggest that excessive PS consumption may alter the differentiation trajectory of intestinal stem cells (ISCs), possibly via the down-regulation of Notch signaling. These results offer important insights into the potential health risks associated with PS exposure.

**Abstract:**

Potassium sorbate (PS) is a preservative widely used in the food, pharmaceutical, and cosmetics industries. Improper and careless use of PS can lead to various health issues and potential environmental problems. *Drosophila* is capable of making rapid and sensitive responses to stress or other stimuli. Here we utilized *Drosophila* as a model organism to evaluate the potential toxicity of PS. Our study revealed that PS ingestion reduced the lifespan and fecundity of *Drosophila*. In addition, excessive PS ingestion led to cell apoptosis and ROS accumulation in the midgut. Furthermore, PS intake also enhanced the mitophagy of midgut cells. Strikingly, PS affected the cell differentiation progression as well, leading to the production of more enteroendocrine (EE) cells. We further demonstrated that the expression of notch (N), a vital player in intestinal stem cell (ISC) differentiation, was down-regulated in the midgut. This indicates that the differentiation progression was affected potentially by repressing the N expression.

## 1. Introduction

Food additives are substances to improve the flavor, taste, texture, appearance, or other qualities of processed food. Till now, they have been widely used all around the world. Individuals consume a substantial quantity of food additives annually [1]. Obviously, the use of food additives offers undeniable benefits in terms of food preservation and palatability, but it is also important to consider the health risks they might pose and the potential bioaccumulation in the natural food chain [2], which may perturb the ecological environment [3]. Indeed, numerous studies have linked food additives to the development of intestinal diseases [4], disruption of gut barrier function [5], initiation of immune responses, and induction of inflammatory reactions leading to tissue damage and associated gastrointestinal and metabolic disorders [6]. Thus, there is a need to carefully assess the safety of food additives.

The elevation of the toxicity of food addition should not be only concentrated on the majority of the population but should also include vulnerable individuals due to their health status or dietary patterns. It is advisable to conduct these assessments using laboratory animals. *Drosophila* is a valuable model organism for investigating biological responses due to its experimental tractability [7] and genetic similarity to humans [8,9]. Moreover, *Drosophila* exhibits rapid and sensitive responses to stress or other stimuli, which is vital for maintaining homeostasis and adapting to environmental changes [10]. This feature, combined with their advantages for biological study, makes them an invaluable model for studying the impact of food additives on organism health.

The digestive tract is the first internal organ where food additives come into contact with an organism. Therefore, it is reasonable to directly detect the physiological and histological changes of the digestive tract after ingestion of potential toxic substances [11]. The midgut is the second-largest organ of *Drosophila* and serves as the major place for food digestion and nutrient absorption [12]. The *Drosophila* midgut is composed of various cell types performing its diverse functions. Among them, enterocytes (ECs) are large polyploid and terminally differentiated cells that secrete digestive enzymes and are involved in nutrient absorption and transport [13]. Enteroendocrine cells (EEs) are implicated in the secretion of gut hormones, which are crucial for maintaining metabolic homeostasis [14]. Both of these two types of cells originate from the intestinal stem cells (ISC) [15]. Notch (N) signaling is a pivotal switch regulating the self-renewal and differentiation of ISC [16]. Repression of notch results in decreased ECs differentiation and elevated EEs number [17]. This regulatory mechanism underscores the complexity of cellular differentiation within the *Drosophila* midgut and its relevance to studying the impact of food additives on digestive health.

Preservatives are a category of the important food additives that are widely used in the food industry for inhibiting microbial growth. For example, in fruit flies, propionic acid is commonly added to fruit fly diets to prevent spoilage [18]. Similarly, various antimicrobial agents, such as potassium sorbate (PS) [19], sodium benzoate (SB) [20], sorbic acid, and diet antimicrobial agents (DAA) [21], are employed in artificial diets for insects reared in the laboratory to control microbial growth. Among these, PS has been prevalently used as a food preservative across a variety of foodstuffs [22]. It has raised concerns due to its potential health effects. A previous study has shown that PS exhibits mutagenic or genotoxic effects in vitro [23], leading to significant increases in chromosomal aberrations and sister chromatid exchanges in lymphocytes [24,25]. In addition, it has been observed that PS shortened the longevity and reduced the percentage of survival of *Drosophila* [26]. Furthermore, PS can alter the diversity and composition of zebrafish gut microbiota and subsequently impact the zebrafish’s immune system [27]. Additionally, continuous PS intake in mice also changed the abundances of gut microbiota and caused infiltration of inflammatory cells in the liver [28]. These findings warrant further investigation into the potential detrimental effects of PS on organisms.

In this study, we examined the physiological and intestinal effects of PS, utilizing *Drosophila* as a model organism. We assessed the impact of PS on various physiological parameters, including fly lifespan, fecundity, cell survival, and cell mitophagy in the midgut, revealing that excessive PS ingestion significantly alters most of these parameters. Our findings further demonstrate that excessive PS intake affects ISCs differentiation trajectory, potentially by down-regulating notch signaling. This research contributes to a deeper understanding of the potential health risks associated with PS consumption.

## 2. Materials and Methods

### 2.1. Drosophila Stocks

The following stocks were used in this study: gut (c601)-Gal4 (BS30844), UAS-mito-QC (BS91640, mitophagy reporter), UAS-CD8-RFP (BS27391), and notch (N)-GFP (BS30729) lines were purchased from Bloomington Stock Center. Esg > GFP (TB00044) flies were obtained from Tsinghua University Research Center. The W1118, tub-Gal80^ts^, UAS-GFP, puc-lacZ fly lines were obtained from Prof. Jie Shen. All flies were raised on standard cornmeal medium at 25 °C. Adult flies that enclosed within one day were then transferred to medium supplemented with Potassium Sorbate (PS) (Wanbang international company) at final concentrations: 0.05%, 0.1%, 0.5%, and 5%. Control groups continued to be raised on the standard medium without PS supplementation. The medium was refreshed every 5 days.

### 2.2. Immunochemistry Staining

Adult midguts were dissected in PBS and fixed in 4% formaldehyde solution for 40 min. Following fixation, samples were rinsed four times with PBT and incubated for 1 h on a rocking shaker in fresh PBT. The tissues were then incubated with the primary antibody overnight at 4 °C. The tissue were rinsed four times with PBT and then washed in PBT for 30 min. After that, they were incubated at 25 °C for 1 h for secondary antibody labeling. Finally, samples were rinsed with PBT and subjected to further analysis. The primary antibody used in this study was mouse anti-β-Gal (1:200; Promega, Z3788, Madison, WI, USA). Secondary antibody was goat anti-mouse DyLight 549 (1:200; Agrisera, Vannas, Swedish). The midguts were mounted on glass slides and images were collected by an inverted Fluorescence Microscope (EVOS FL, Life Technologies, Carlsbad, CA, USA) or a confocal laser scanning microscope (LSM 880, Carl Zeiss, Oberkochen, Germany).

### 2.3. Lifespan Assay

Within 1 day of eclosion, 600 W1118 flies were collected and randomly divided into four groups. Each group consisting of 150 flies was further divided into 10 replicates. The flies were cultured on either standard medium (control) or PS medium (0.05%, 0.5%, 1%, 5%). Fly survival was monitored daily until all individuals had died.

### 2.4. Fecundity Assay

10 virgin females mated with 10 fresh males on standard or PS medium in a vial for 7 days. Medium was exchanged daily, and egg number was counted at 24-h intervals. Larval development was monitored until pupation.

### 2.5. Fly Gut Acidity Assay

To examine the gut acidity, flies collected within 24 h of eclosion were fed with either standard medium or PS medium for 7 days. Following this feeding period, flies were exposed to a yeast paste containing 0.5 g of dry yeast and 1 mL of 0.15% Bromophenol Blue dye (Sigma-Aldrich, Missouri, USA) for 8 h. Guts were then dissected in PBS and analyzed immediately. Images were captured using an Olympus mvx10 microscope (Olympus, Tokyo, Japan).

### 2.6. Trypan Blue Staining

Guts were dissected in PBS and then washed repeatedly, and stained with 0.02% Trypan blue solution for 15 min. After further washing for 15 min in PBS, samples were imaged using an Olympus mvx10 fluorescence microscope.

### 2.7. ROS Detection

Adult guts were dissected in PBS and subsequently incubated in 100 μL of cell culture medium with 0.4 μL ROS reagent (CM-H2DCFDA). After incubation in darkness for 5 min, the tissue was washed four times with PBS, and then fixed in 4% paraformaldehyde for 20 min. After fixation, the guts were washed again four times with PBS and mounted on glass slides for observation.

### 2.8. Notch Signaling Detection

Adult guts were dissected in PBS and subsequently embedded in 30% glycerol. Then the samples were mounted on glass slides and observed on an inverted Fluorescence Microscope (EVOS FL, Life Technologies, Carlsbad, CA, USA).

### 2.9. Real-Time Quantitative PCR

The midgut RNA was extracted using RNAiso Plus (TaKaRa, Japan). 500 ng of total RNA was used to synthesize first-strand cDNA by using M-MLV reverse transcriptase (Takara Bio, Kusatsu, Japan). RT-qPCR was carried out using SYBR^®^Premix EX Taq™ (TaKaRa, Kusatsu, Japan) on a Bio-Rad system (Bio-Rad Laboratories, Hercules, USA). *Rp49* and *Gapdh* were used as internal references gene (Table S1). The data are presented as means ± SEM of three independent biological replications.

### 2.10. Statistical Analysis

Statistical analyses were performed using GraphPad Prism 8.0.2. Results are expressed as mean ± standard error (SEM). Statistical significance between two means was assessed using the two-tailed Student’s *t*-test. A *p*-value of less than 0.05 was considered statistically significant for all analyses.

## 3. Results

### 3.1. PS Ingestion Reduced the Lifespan and Fecundity of Drosophila

**Figure 1:**
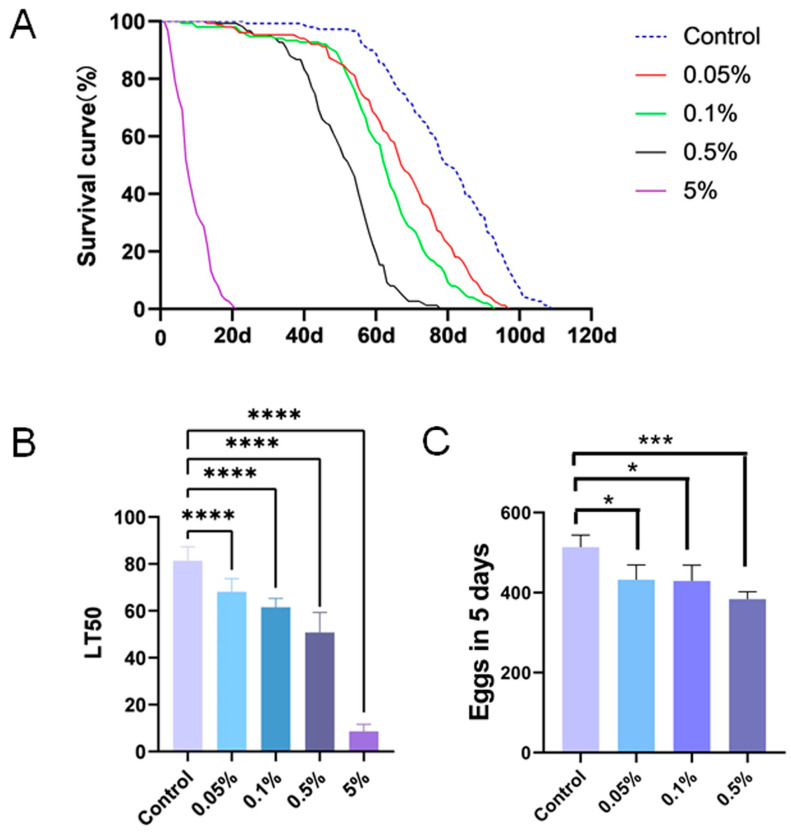
The impact of PS intake on the lifespan and fecundity of *Drosophila*. (**A**) PS intake led to a reduction in *Drosophila* longevity. (**B**) The LT50 of flies fed with PS. (**C**) The overall quantity of eggs laid over a span of 5 days. *p* ≤ 0.05 was considered significant (*), *p* ≤ 0.001 (***), and *p* ≤ 0.0001 (****).

Previous studies have demonstrated the toxicity of PS to organisms [25]. Given the growing concern about health impacts of PS, we sought to explore *Drosophila* as a model organism to elucidate the effects of PS in a living organism. To begin with, we investigated its potential effects on the lifespan of *Drosophila*. PS was incorporated into the *Drosophila* medium at varying concentrations of 0.05%, 0.1%, 0.5%, and 5%. The medium was replaced every 5 days, and the number of deceased flies was recorded daily until all the flies died. This daily monitoring allowed us to accurately assess the impact of PS on *Drosophila* longevity. Our results revealed that the ingestion of PS significantly shortened the lifespan of *Drosophila* compared to the control group (Figure 1A). Moreover, the median lethal time (LT50) for each PS concentration was significantly lower than that of the control group (Figure 1B). Flies fed 5% of PS exhibited an extremely reduced lifespan compared to the control group. The longest-lived flies in the 5% PS group only survived for less than 20 days, and the mean LT50 is 8.6 days; thus, we opted to use 0.05%, 0.1%, and 0.5% concentrations for the following studies. These findings underscore the detrimental effects of PS on the longevity of *Drosophila*.

Fecundity is an important physiological indicator of *Drosophila* [29]. We investigated the effects of different concentrations of PS on the fecundity of *Drosophila*. We cultured 10 virgin females and 10 virgin males on PS food at concentrations of 0.05%, 0.1%, and 0.5% for 7 days. From the 8th day, these 20 flies were transferred to normal medium for mating and egg-laying. Our results indicated a notable decrease in egg laying of flies cultured on medium supplemented with 0.05%, 0.1%, and 0.5% PS compared to the control group, with the most pronounced effect observed at the 0.5% PS group (Figure 1C). This indicates PS ingestion impairs the fecundity of *Drosophila*.

### 3.2. The PS Ingestion Disrupted Cell Membrane Integrity but Did Not Alter Midgut pH

**Figure 2:**
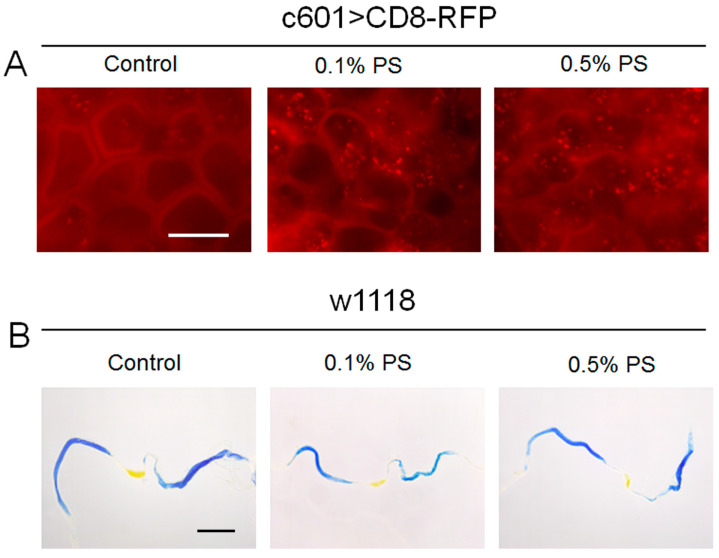
PS impacts the cell membrane of the midgut, while not affecting the pH. (**A**) UAS-CD8-RFP was driven by c601-Gal4 to mark the cell membrane of the midgut. The cell membrane in the midguts of flies fed with 0.1% and 0.5% PS appears fuzzy, specifically in the midguts of 0.5% PS-fed flies. (**B**) An acidic area, indicated by the yellow color, is noticeable in the center of control midgut, while other regions appeared blue. BPB staining demonstrated that the midgut’s pH was not visibly influenced by 0.1% and 0.5% PS ingestion.

The integrity of the cell membrane and the morphology of cells are informative indicators of cellular physiology [30]. Next, we investigated the impact of PS on the cell membrane integrity of the midgut. In our study, the midgut cell membrane was labeled with CD8-RFP, which was driven by the gut-Gal4. We found that 0.1% PS treatment compromised cell membrane integrity. Furthermore, the higher concentration of 0.5% PS exhibited the more severe damage, with apparent disruption of cell membrane integrity observed (Figure 2A). The disruption of cell membrane structures underscores the potential for adverse effects on cellular function and health.

Midgut pH is mildly acidic to neutral in some groups of insects [31]. The midgut pH in insects is thought to be a result of adaptation to a particular diet [32]. We sought to detect whether midgut pH is influenced by PS ingestion using a Bromophenol Blue dye (BPB). After 7 days of rearing, both the control and treated groups of flies were fed with a yeast paste containing 0.5 g of dry yeast and 1 mL of 0.15% BPB for 8 h. Then the midgut was dissected and detected. Consistent with the previous report, our results showed that an acidic region displaying yellow color was situated in the middle segment of the control midgut, while the other regions appeared blue [33]. Importantly, we observed no significant changes in midgut pH in flies fed with 0.1% PS and 0.5% PS (Figure 2B). This suggests that midgut pH was not notably impacted by the intake of PS.

### 3.3. PS Ingestion Induced Cell Apoptosis in the Midgut

**Figure 3:**
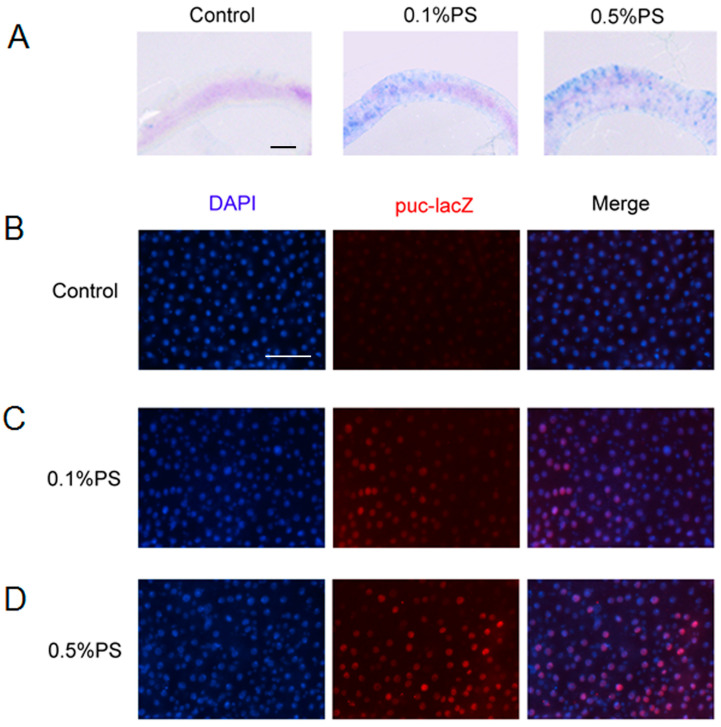
PS ingestion resulted in cell apoptosis in the midgut. (**A**) The Trypan blue staining exhibited induced cell death in the midguts fed with 0.1% and 0.5% PS. (**B**–**D**) The puc-lacZ staining also demonstrated that cell death was induced by PS ingestion.

Harmful substances typically induce cell apoptosis [34]. We investigated whether PS induced apoptosis in the midgut. Firstly, we performed Trypan Blue assays, which differentiate between living and dead cells by labeling the latter blue while leaving live cells unstained [35]. Our findings revealed that intestinal cells from *Drosophila* treated with 0.1% PS exhibited Trypan Blue staining, indicating a subpopulation of dead cells compared to the control group. Notably, flies treated with 0.5% PS displayed a marked increase in the number of stained cells within the midgut, suggesting a more pronounced induction of apoptosis (Figure 3A). The JNK signaling pathway is a critical regulator of apoptosis and cellular stress responses [36]. To investigate the potential involvement of JNK signaling in PS-induced apoptosis, we employed a puc-lacZ reporter, which serves as a readout for JNK activity in *Drosophila*. Our results revealed an apparent increase of the puc-lacZ-positive cells in the gut of flies treated with 0.1% PS compared to the control group (Figure 3B,C). Evidently, an even more significant increase was observed in flies treated with 0.5% PS (Figure 3B,D). These findings suggest that PS ingestion induced cell apoptosis in a dose-dependent manner.

### 3.4. PS Ingestion Induced Elevated Level of ROS in the Midgut

Reactive oxygen species (ROS) is a key inducer of cell apoptosis and one of the vital parameters for toxicity evaluation [37]. It plays important roles in many homeostatic processes, such as cell signaling, metabolism, and immunity. When the body is stimulated by external stimuli, the excessive accumulation of ROS will destroy the cell homeostasis, leading to oxidative stress damage, cell apoptosis, and mitochondrial dysfunction. These damages can cause various diseases, such as inflammation, aging, and even cancer.

**Figure 4:**
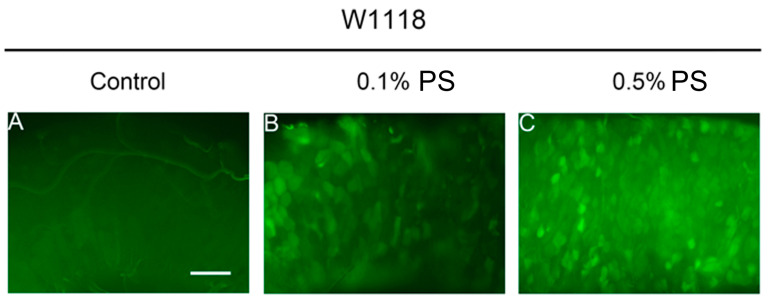
PS ingestion resulted in ROS accumulation in the midgut. (**A**–**C**) The figure shows ROS detection in the midguts of flies fed on standard medium or PS medium (0.1% and 0.5%). Scale bar = 50 μm.

ROS is intricately linked to JNK through mechanisms that influence cellular processes like apoptosis and cell survival [38]. ROS act as signaling molecules that trigger the JNK pathway [39]. Increased ROS levels activate JNK, establishing a feedback loop. In addition, JNK also plays a role in modulating ROS levels within cells. For instance, during oxidative stress, JNK can trigger the expression of genes involved in the antioxidant response, thereby managing ROS levels [40]. To explore whether PS could induce excess ROS production, we stained the dissected midguts of *Drosophila* fed different concentrations of PS with CM-H2DCFDA, a fluorescent ROS-sensing dye. The results showed that 0.1% PS increased ROS levels in the intestine compared to the control group (Figure 4A,B), while 0.5% PS resulted in a significant increase in ROS levels (Figure 4A,C). This suggests that the accumulation of ROS might lead to cell apoptosis in the midgut.

### 3.5. PS Ingestion Enhanced Mitophagy in the Midgut

Mitophagy is a process for the selective removal of damaged or excess mitochondria through autophagy. It plays a crucial role in maintaining mitochondrial homeostasis and normal cellular function [41]. Defects of mitophagy can lead to the accumulation of damaged and dysfunctional mitochondria, contributing to metabolic issues and developmental problems [42]. Mitophagy is a major source of ROS generation [43]. To investigate whether different concentrations of PS induced mitophagy in the midgut, we detected mitophagy in gut cells by using a mito-QC reporter under the control of a gut-Gal4 driver. Mito-QC utilizes mitochondrial-targeted tandem mCherry-GFP fluorescence tags located on the outer mitochondrial membrane [44]. When the damaged mitochondria are delivered to lysosomes, the GFP signal is quenched irreversibly, whereas the mCherry fluorescence is resistant to the acidic environment of autolysosomes. Thus, the degree of mitophagy can be quantified by evaluating the quantity of red-only puncta in cells [45].

**Figure 5:**
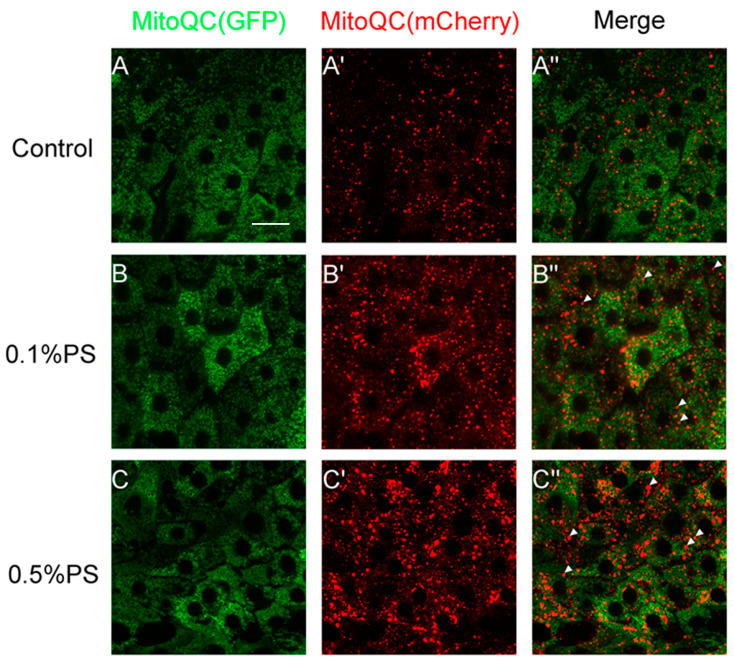
PS ingestion induced mitophagy in the midgut. (**A**–**C**) The midguts from flies that were fed on 0.1% and 0.5% PS medium exhibited more severe mitophagy when compared with the control group. White arrows indicate the red-only punctas.

Our results showed a notable increase in red-only puncta in intestinal cells after 7 days of 0.1% PS ingestion (Figure 5A,B). On the other hand, flies fed a diet with 0.5% PS displayed an even greater number of red-only puncta, which were also larger in size (Figure 5A,C). These results indicate a dose-dependent enhancement of mitophagy activation.

**Figure 6:**
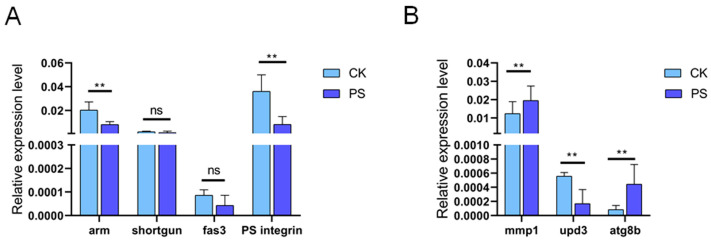
The gene expression levels in the midguts of flies fed with standard medium or PS medium. (**A**) The expression levels of genes related to cell membrane integrity. (**B**) The expression levels of genes related with cell apoptosis or mitophagy. All data are reported as means ± SEM of three independent biological replications. The asterisks indicate significance differences between the control and PS-fed groups (*p* ≤ 0.01, **), while “ns” means no statistically significant differences.

To further explore the underlying mechanism of the above alterations, we performed RT-PCR experiments to identify any potential candidate genes that may have induced these changes. We focused on genes implicated in cell membrane integrity maintenance, including *armadillo* (*arm*), *shortgun*, *fasciclin III* (*fas3*), and *PS integrin* [46,47]. The results revealed significant down-regulation of *arm* and *PS integrin* expression, while *shotgun* and *fas3* levels remained largely unchanged in the midgut of *PS ingestion* flies (Figure 6A). This implies that the integrity of the cell membrane could possibly be linked with the diminished expression levels of *arm* and *PS integrin*. In addition, we also detected the expression of *upd3*, *Matrix Metalloprotease 1* (*MMP1*), and *atg8b*. Our results indicated an up-regulation of *MMP1* and *atg8b*, while we observed a decrease in *upd3* expression. *MMP1*, acting as an indicator of JNK signaling [48], and *atg8b*, which is implicated in mitophagy [49], were both elevated (Figure 6B). These findings suggest that excessive intake of PS triggers a variety of genes associated with cellular apoptosis, cell membrane integrity maintenance, and mitophagy.

### 3.6. PS Ingestion Altered ISC Differentiation Trajectory

**Figure 7:**
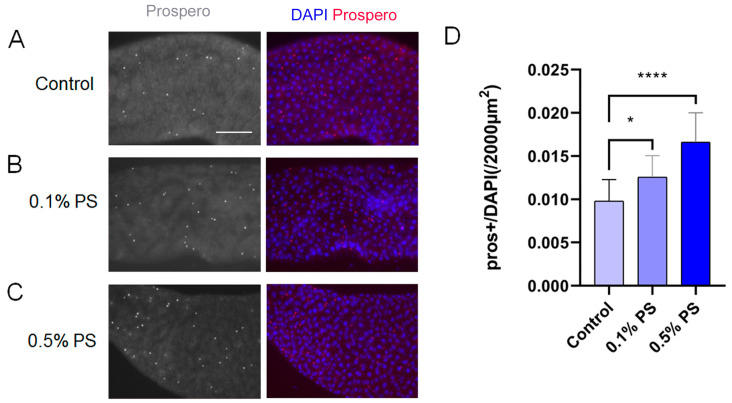
PS ingestion alters ISCs differentiation trajectory. (**A**–**C**) EE cells in the midgut from flies fed with 0.1% and 0.5% PS medium were labeled by Prospero antibody staining. (**D**) Quantitative analysis of the Prospero antibody-labeled EE cells, the number of EE cells were increased in the midguts of the flies fed with PS. All data are reported as means ± SEM of three independent biological replications. *p* ≤ 0.05 was considered significant (*) and *p* ≤ 0.0001 (****).

In *Drosophila* midgut, the intestinal stem cells (ISC) terminally differentiated into absorptive enterocyte (EC) and secretory enteroendocrine (EE) cells [15]. This process of differentiation is tightly regulated. We labeled the EE cells with an antibody against Prospero, which serves as a marker for EE cells. To examine if the cell differentiation in midguts was affected by PS ingestion, we quantified the Prospero-positive EE lineage. Our results showed an increased proportion of Prospero-positive cells in the midgut of 0.1% PS-fed flies (Figure 7A,B,D), with a pronounced increase observed in the 0.5% PS group (Figure 7A,C,D). This suggests that a larger number of ISCs are differentiating into EE cells compared to the control group.

### 3.7. Decreased Notch Expression Was a Potential Cause of the Observed Changes in Cell Differentiation

**Figure 8:**
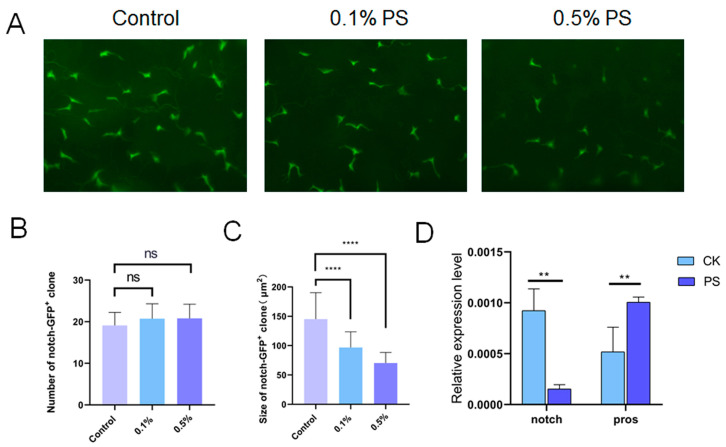
PS ingestion led to the reduction of Notch signaling. (**A**) The N-GFP in the midgut of flies fed with the standard medium or PS medium (0.1% and 0.5%). Scale bar = 50 μm. (**B**) PS ingestion did not significantly influence the number of N-GFP clones. (**C**) PS ingestion notably reduced the size of N-GFP clones. (**D**) RT-qPCR result showed that the *notch* expression was down-regulated in midgut of 0.5% PS-fed flies. All data are reported as means ± SEM of three independent biological replications. The asterisks indicate significance differences between the control and PS-fed groups (*p* ≤ 0.01, **; *p* ≤ 0.0001 ****), while “ns” means no statistically significant differences.

A range of studies have shown that notch (N) signaling plays a key role in the control of ISC differentiation [16]. Based on these findings, we sought to explore if there were any alterations in expression of notch (N) following the ingestion of PS. We used an N-GFP stock to monitor the expression activity of N in the midgut. To avoid any possible alteration in signaling distribution due to fixation, we opted to directly observe the live tissue (Figure 8A). Our results revealed no significant difference in the number of N-GFP clones present in the midguts of flies fed with either 0.1% or 0.5% PS compared to the control group (Figure 8B). However, we noted an observable decrease in the size of N-GFP clones in both 0.1% and 0.5% PS-fed flies (Figure 8C). This indicates that the PS ingestion potentially inhibits N signaling within the midgut.

Furthermore, we conducted RT-qPCR experiments to further determine whether the expression of N was down-regulated. The results confirmed a decrease in N expression (Figure 8D). Together, we deduced that PS ingestion increases the enteroendocrine (EE) cell population in the midgut, potentially by repressing the N expression.

## 4. Discussion

Potassium sorbate (PS) is a white and odorless potassium salt of sorbic acid, widely used in the pharmaceutical, cosmetic, and food industries [22]. As a potent food additive, PS is frequently employed in processed food products. Several studies have shown that PS has deleterious effects on the health of an organism [23]. For example, PS has the potential to induce chromosomal aberrations and sister chromatid exchanges [24]. Additionally, PS can result in notable DNA strand breaks, even when administered at low concentrations [25]. Other studies have demonstrated that PS possesses the ability to stimulate mutagenic and genotoxic effects in Chinese Hamster ovary cells [23]. The pathogenesis of many human diseases remains elusive, and harmful substances in food are potential contributors to these increasing miscellaneous diseases. Therefore, a careful assessment of the toxicity of synthetic chemicals incorporated into foodstuffs is imperative. In addition, considering that processed food is also consumed by both domesticated animals and urban wildlife [50], PS may consequently pose a perturbation to the balance of the ecosystem to some extent.

A comprehensive assessment of the toxicity of PS to organism health is required. Our study demonstrates that intake of PS significantly diminished the longevity of *Drosophila*. The intestine serves as a direct interface for interaction between food and organisms. We evaluated the detrimental effects of PS on the *Drosophila* midgut. We found that excessive PS ingestion not only disrupted the cell membrane but also triggered an accumulation of ROS, which are known to play a pivotal role in inducing cell apoptosis [37]. To further assess the impact of PS on midgut cell apoptosis, we observed an increase in cells marked with puc-lacZ and Trypan blue staining, indicating elevated apoptosis. Additionally, some cells exhibited abnormal nuclear morphology, with nuclei boundaries becoming irregular and blurred. To further assess the impact of PS on midgut cell apoptosis, we observed that the exposure to excess PS resulted in significant midgut cell damage. Accordingly, we detected the cell membrane-associated genes *arm* and PS *integrin* were down-regulated. Moreover, JNK indicator gene MMP1 and mitophagy-related gene atg8b [51] were up-regulated.

Impaired mitochondria can be selectively eliminated through mitophagy. Dysregulation of mitophagy is linked to the pathogenesis of neurodegenerative diseases and metabolic disorders [41]. Our findings demonstrated a significant up-regulation of mitophagy upon exposure to PS, suggesting that PS may disrupt the homeostasis of intracellular processes.

In the midgut, ISCs serve as a stem population, giving rise to terminal differentiated EE and EC cells, and are crucial for maintaining midgut homeostasis through self-renewal [15]. We observed that PS exposure affected the differentiation processes of the ISCs. This dysregulation of ISCs activity could consequently impair midgut self-renewal and tissue homeostasis, potentially contributing to numerous diseases such as inflammatory bowel or metabolic syndrome. Notch plays an instructive role in the regulation of ISC differentiation [16]. Interestingly, our findings revealed a reduction in notch signaling activity under PS exposure conditions. We therefore deduced that the aberrance in ISC differentiation might be a consequence of down-regulated notch signaling due to dietary PS ingestion.

This study demonstrates that excessive ingestion of PS affects various physiological parameters in *Drosophila* and lower concentrations of PS are relatively safe. Given that PS is widely used as a classic food preservative, it is crucial to carefully consider its concentration in food. Individuals should balance their dietary habits and health status when consuming foods containing PS. Additionally, PS is employed as a preservative in the feed of various insects [19]. Due to differing tolerances of additives among different insect species, the safe dosage of PS in insect diet may vary. The concentrations used in this study can serve as a reference for determining appropriate levels.

## 5. Conclusions

PS is a synthetic preservative long-considered harmless. It has raised concerns due to its DNA damage and other deleterious effects on organisms. Our study explored the PS’s impact on *Drosophila* lifespan and reproduction, revealing detrimental effects on these aspects at high concentrations. PS exposure is sufficient to induce intestinal cell damage, alter exosome secretion patterns, and enhance mitophagy. Additionally, PS skewed ISC differentiation patterns towards EE cells, potentially via down-regulating the *notch* expression. These findings highlight the need for careful reevaluation of PS safety and provide valuable insights into its potential toxic mechanisms.

## Data Availability

All the data generated in this work were provided in the article.

## Supplementary Materials

The following supporting information can be downloaded at: https://www.mdpi.com/article/10.3390/insects15090703/s1, Table S1. Primers for RT-qPCR amplification. This table lists all the primers used in the RT-qPCR experiments conducted in this study.
