# Supplementary material for: Toxicity Evaluation of Potassium Sorbate In Vivo with Drosophila Melanogaster
Source: Insects. 2024 Sep 14;15(9):703. doi: 10.3390/insects15090703 (PMC11432522; doi:10.3390/insects15090703)
Supplement: Supplementary file 1 [file insects-15-00703-s001.zip › insects-3159988-supplementary.pdf]

Table S1. Primers for RT-qPCR amplification.

Gene	Primer	Primer Sequences
rp49	Forward	GACAGTATCTGATGCCCAACA
	Reverse	CTTCTTGGAGGAGACGCCGT
Gapdh	Forward	GTGAACGGCCAGAAGATCAC
	Reverse	GGACTCCACCACATACTCGG
atg8b	Forward	AGTTCTACTTTCTCATCCGC
	Reverse	AGTTCTACTTTCTCATCCGC'
mmp1	Forward	GGACACCGACCTGACCTT
	Reverse	TGGAACAGATTGGTGCCTC
upd3	Forward	CCACCAATGCGGACAAG
	Reverse	ATTCAGACGGGGCAGGAA
arm	Forward	GCTTAAACTACGGACTATCGG
	Reverse	CTGTGAACCAGTGGTGGC
shortgun	Forward	CAGACCGCACCTACCAAC
	Reverse	CGAATGCAACCGAAGAAA
fas3	Forward	ATCGTTGGCATTGTGGTC
	Reverse	TCGCTGGTTTCGTTTGTG
PS integrin	Forward	CGTCGGCTGTTGGTCTTC
	Reverse	TGTACTCGCCCTTGGGAC
notch	Forward	GTGCGATAAGCGAGGTTG
	Reverse	CACGGATTGATGCCCAGT
